# Supplementary material for: Treatment of Heart Failure Patients with Anxiolytics Is Associated with Adverse Outcomes, with and without Depression
Source: J Clin Med. 2020 Dec 7;9(12):3967. doi: 10.3390/jcm9123967 (PMC7762354; doi:10.3390/jcm9123967)
Supplement: Supplementary file 1 [file jcm-09-03967-s001.pdf]

Supplementary Table 1. Demographics and clinical characteristics of patients with heart failure according to treatment with anxiolytics, depression or both.

**Variable**	**None (N=4160)**	**Anxiolytic (N=445)**	**Depression (N=1180)**	**Depression and Anxiolytic (N=508)**	**Total (N=6293)**	**P Value**
Age (Years)	73 (64-82)	78 (67-85)	81 (72-88)	81 (71-87)	76 (66-84)	<0.001
Men	2436 (59)	228 (51)	489 (41)	188 (37)	3341 (53)	<0.001
Social Status (living with spouse)	2165 (53)	206 (47)	411 (35)	174 (34)	2956 (48)	<0.001
NYHA Class III/IV	1091 (33)	133 (40)	374 (44)	169 (47)	1767 (37)	<0.001
HF Type						
Reduced ejection fraction	1194 (29%)	126 (28%)	299 (25%)	124 (24%)	1743 (28%)	
Preserved ejection fraction	1479 (36%)	175 (39%)	513 (43%)	226 (44%)	2393 (38%)	<0.001
Not-specified	1487 (36%)	144 (32%)	368 (31%)	158 (31%)	2157 (34%)	
Diabetes mellitus	2232 (54)	211 (47)	620 (53)	269 (53)	3332 (53)	0.09
Hypertension	3294 (79)	382 (86)	1032 (87)	455 (90)	5163 (82)	<0.001
Hyperlipidemia	3672 (88)	403 (91)	1077 (91)	463 (91)	5615 (89)	0.008
Ischemic Heart Disease	2736 (66)	310 (70)	759 (64)	316 (62)	4121 (65)	0.08
Prior Myocardial Infarction	1790 (43)	199 (45)	466 (39)	192 (38)	2647 (42)	0.02
Prior coronary bypass surgery	83 (2)	7 (2)	18 (2)	4 (0.8)	112 (2)	0.21
Atrial fibrillation	1454 (35)	186 (42)	481 (41)	211 (42)	2332 (37)	<0.001
Prior Stroke/ transient ischemic attack	769 (18)	95 (21)	399 (34)	152 (30)	1415 (22)	<0.001
Peripheral vascular disease	506 (12)	64 (14)	210 (18)	95 (19)	875 (14)	<0.001
Chronic obstructive lung disease	828 (20)	113 (25)	276 (23)	116 (23)	1333 (21)	0.004
Charlson Score	6.0 (4.0-7.0)	6.0 (5.0-8.0)	7.0 (6.0-8.0)	7.0 (5.0-8.0)	6.0 (5.0-7.0)	<0.001
Malignancy	780 (19)	120 (27)	280 (24)	132 (26)	1312 (21)	<0.001
Dementia	282 (7)	47 (11)	314 (27)	152 (30)	795 (13)	<0.001

Dialysis	205 (5)	26 (6)	51 (4)	18 (4)	300 (5)	0.31
Body mass index (kg/m^2)	29 (26-33)	28 (25-32)	29 (25-33)	30 (25-33)	29 (25-33)	0.009
Pulse (beats per minute)	72 (65-80)	72 (64-80)	72 (65-80)	72 (64-80)	72 (64-80)	0.18
Systolic blood pressure (mmHg)	127 (118-138)	129 (119-139)	128 (117-139)	128 (116-140)	128 (118-139)	0.75
Diastolic blood pressure (mmHg)	72 (65-80)	72 (63-79)	70 (63-79)	70 (64-80)	72 (65-80)	0.010
**Laboratory Data**	**None (N=4160)**	**Anxiety (N=445)**	**Depression (N=1180)**	**Depression and Anxiety (N=508)**	**Total (N=6293)**	**P Value**
Creatinine (mg/dL)	1.0 (0.8-1.3)	1.0 (0.8-1.3)	1.0 (0.8-1.3)	1.0 (0.8-1.3)	1.0 (0.8-1.3)	0.86
Estimated glomerular filtration rate (mL/min per 1.73m^2)*	73 (52-95)	69 (49-91)	66 (48-89)	66 (48-86)	71 (51-93)	<0.001
Urea (mg/dL)	44 (33-60)	46 (34-64)	48 (35-67)	48 (34-64)	45 (34-62)	<0.001
Urine Albumin / Creatinine ratio	37 (12-154)	37 (13-137)	34 (14-153)	35 (13-136)	35 (13-148)	0.99
Sodium (mEq/L)	140 (138-142)	140 (138-142)	140 (138-142)	140 (138-142)	140 (138-142)	0.75
Potassium (mEql/L)	4.6 (4.3-5.0)	4.6 (4.3-4.9)	4.6 (4.3-4.9)	4.6 (4.3-4.9)	4.6 (4.3-4.9)	0.12
Hemoglobin (g/dL)	13 (12-14)	13 (11-14)	13 (11-14)	12 (11-14)	13 (12-14)	<0.001
White blood count (x10^9/L)	7.3 (6.1-8.9)	7.3 (5.9-8.8)	7.3 (6.0-8.7)	7.1 (6.0-8.8)	7.3 (6.0-8.8)	0.54
Red Cell Distribution Width (%)	15 (14-16)	15 (14-16)	15 (14-16)	15 (14-16)	15 (14-16)	<0.001
Glucose (mg/dL)	108 (95-135)	105 (93-127)	103 (92-129)	103 (92-126)	106 (94-133)	<0.001
Hemoglobin A1c (%)	6.2 (5.6-7.2)	6.0 (5.5-6.8)	6.0 (5.6-7.0)	6.0 (5.5-6.8)	6.1 (5.6-7.1)	<0.001
Uric Acid (mg/dL)	6.3 (5.1-7.5)	6.2 (4.9-7.5)	6.2 (5.1-7.5)	6.2 (5.0-7.6)	6.2 (5.1-7.5)	0.77
TSH (mIU/L)	2.2 (1.4-3.3)	2.3 (1.5-3.6)	2.2 (1.5-3.3)	2.4 (1.5-3.6)	2.2 (1.5-3.3)	0.12
Iron (µg/dL)	60 (44-80)	57 (42-76)	57 (41-76)	56 (41-74)	59 (43-79)	<0.001
Transferrin (mg/dL)	252 (220-293)	249 (211-292)	241 (209-277)	244 (210-285)	249 (216-289)	<0.001
Transferrin Saturation (%)	17 (12-23)	16 (11-23)	17 (12-23)	16 (12-22)	17 (12-23)	0.57
Ferritin (ng/ml)	82 (37-166)	76 (37-162)	78 (40-170)	78 (36-166)	80 (38-166)	0.91
Calcium (mg/dL)	9.3 (9.0-9.6)	9.2 (8.9-9.5)	9.2 (8.9-9.5)	9.2 (8.9-9.5)	9.3 (9.0-9.6)	<0.001
Phosphorus (mg/dL)	3.5 (3.1-3.9)	3.5 (3.1-3.9)	3.5 (3.2-3.9)	3.5 (3.2-4.0)	3.5 (3.1-3.9)	<0.001

Magnesium (mg/dL)	2.1 (1.9-2.3)	2.1 (1.9-2.3)	2.1 (2.0-2.3)	2.2 (1.9-2.3)	2.1 (1.9-2.3)	0.08
Triglycerides (mg/dL)	120 (88-171)	119 (92-164)	121 (92-166)	123 (89-174)	121 (89-169)	0.78
Low-density lipoprotein (mg/dL)	82 (65-105)	86 (66-110)	83 (66-108)	85 (64-106)	83 (65-106)	0.12
Albumin (g/dL)	4.0 (3.7-4.2)	3.9 (3.7-4.1)	3.8 (3.5-4.1)	3.8 (3.5-4.1)	3.9 (3.7-4.2)	<0.001
C-Reactive Protein (mg/dL)	0.5 (0.2-1.4)	0.6 (0.2-2.0)	0.7 (0.2-1.7)	0.6 (0.2-1.4)	0.6 (0.2-1.5)	0.07
Alanine transaminase (IU)	16 (12-22)	16 (11-23)	14 (10-20)	15 (11-20)	16 (12-22)	<0.001
Alkaline Phosphatase (IU)	87 (70-111)	89 (70-112)	90 (73-113)	88 (72-110)	88 (71-111)	0.04
Total Bilirubin (mg/dL)	0.6 (0.5-0.8)	0.6 (0.4-0.7)	0.5 (0.4-0.7)	0.5 (0.4-0.7)	0.6 (0.4-0.8)	<0.001
Gamma-glutamyltransferase (IU)	25 (18-40)	27 (18-50)	25 (17-44)	27 (18-49)	25 (17-42)	0.02
**Medication**	**None (N=4160)**	**Anxiety (N=445)**	**Depression (N=1180)**	**Depression and Anxiety (N=508)**	**Total (N=6293)**	**P Value**
ACE-I/ARB/ARNI	3289 (79)	362 (81)	867 (73)	385 (76)	4903 (78)	<0.001
Beta blockers	3114 (75)	348 (78)	853 (72)	382 (75)	4697 (75)	0.09
Spironolactone	1444 (35)	170 (38)	422 (36)	200 (39)	2236 (36)	0.12
Furosemide	2577 (62)	312 (70)	844 (72)	391 (77)	4124 (66)	<0.001
Thiazide	588 (14)	53 (12)	164 (14)	62 (12)	867 (14)	0.42
Digoxin	232 (6)	31 (7)	86 (7)	34 (7)	383 (6)	0.12
Amiodarone	671 (16)	87 (20)	146 (12)	82 (16)	986 (16)	0.001
Aspirin	2388 (57)	252 (57)	603 (51)	274 (54)	3517 (56)	0.001
New oral anticoagulants**	1014 (24)	142 (32)	358 (30)	152 (30)	1666 (26)	<0.001
Vitamin K antagonists	626 (15)	62 (14)	152 (13)	75 (15)	915 (15)	0.3

Data is presented as median (inter-quartile range) for continuous variables and counts (percentages) for categorical variables. P value by the Kruskal Wallis Test for continuous variables and the Chi-Square Test for categorical variables.

Diabetes mellitus defined as fasting plasma glucose ≥ 126 mg/dL or glucose lowering treatment, hypertension as blood pressure > 140/90 mmHg measured on several occasions or anti-hypertensive treatment and hyperlipidemia as low density lipoprotein > 130 mg/dL, fasting serum triglycerides > 200 mg/dL or lipid lowering treatment.

* Estimated Glomerular Filtration Rate was calculated using the modified Modification of Diet in Renal Disease (MDRD) equation (175 * serum creatinine$^{-1.154}$ * age$^{-0.203}$. For females a correction factor is used multiplying by 0.742.)

** Dabigatran, Rivaroxaban or Apixaban.

Supplemental Table 2. Hazard ratios for clinical outcome according to anxiety and depression status as time-dependent variables by Cox regression analysis.

	**Univariable**		**Multivariable**		**Multivariable and Drugs**	
	**Hazard Ratio (95% CI)**	**P-value**	**Hazard Ratio (95% CI)**	**P-value**	**Hazard Ratio (95% CI)**	**P-value**
Main Analysis						
Death						
Anxiolytics	1.56 (1.39-1.76)	<0.001	1.48 (1.30-1.69)	<0.001	1.55 (1.36-1.77)	<0.001
Depression	1.81 (1.63-2.01)	<0.001	1.45 (1.29-1.63)	<0.001	1.42 (1.26-1.60)	<0.001
Death and cardiovascular hospitalization						
Anxiolytics	1.23 (1.12-1.34)	<0.001	1.15 (1.05-1.26)	0.003	1.14 (1.04-1.25)	0.005
Depression	1.38 (1.29-1.48)	<0.001	1.24 (1.15-1.34)	<0.001	1.22 (1.13-1.32)	<0.001
Additional Analysis						
Death						
None	1.0 (Reference)		1.0 (Reference)		1.0 (Reference)	
Anxiolytics (Alone)	2.00 (1.70-2.35) <0.001		1.62 (1.36-1.94) <0.001		1.68 (1.41-2.01) <0.001	
Depression (Alone)	2.05 (1.82-2.31) <0.001	<0.001	1.53 (1.33-1.75) <0.001	<0.001	1.49 (1.30-1.71) <0.001	<0.001
Depression and Anxiolytics	2.52 (2.16-2.94) <0.001		2.05 (1.72-2.44) <0.001		2.11 (1.77-2.51) <0.001	
Death and cardiovascular hospitalization						
None	1.0 (Reference)		1.0 (Reference)		1.0 (Reference)	
Anxiolytics (Alone)	1.34 (1.20-1.50) <0.001		1.18 (1.05-1.33) 0.007		1.19 (1.05-1.34) 0.005	
Depression (Alone)	1.44 (1.33-1.55) <0.001	<0.001	1.26 (1.15-1.37) <0.001	<0.001	1.25 (1.15-1.36) <0.001	<0.001
Depression and Anxiolytics	1.57 (1.40-1.77) <0.001		1.39 (1.22-1.59) <0.001		1.35 (1.18-1.54) <0.001	

Data is presented as hazard ratio (95% confidence interval), P value.

Parameters that were included in the multivariable analysis model were age, gender, NYHA functional class, diabetes, hypertension, ischemic heart disease, atrial fibrillation, log-transformed body mass index, log-transformed serum urea levels, square root-transformed estimated glomerular filtration rate, hemoglobin, serum sodium, treatment for anxiety and depression status.

Parameters included in the additional multivariable analyses were the above parameters with the substitution of anxiolytics and depression with a parameter that included either or both.
